# A Giant Enteric Duplication Cyst Presenting as Mechanical Intestinal Obstruction in a Four-Year-Old Child: A Case Report

**DOI:** 10.7759/cureus.111660

**Published:** 2026-06-28

**Authors:** Sophiko Osiashvili, Darejan Kanjaradze, Ekaterine Gozalishvili, Vasil Kareli, Ia Khurtsilava

**Affiliations:** 1 Pediatrics, Petre Shotadze Tbilisi Medical Academy, Tbilisi, GEO; 2 Pediatric Intensive Care Unit, Tbilisi Pediatric Private Clinic, Tbilisi, GEO; 3 Pediatric Emergency Department, Tbilisi Pediatric Private Clinic, Tbilisi, GEO; 4 Pediatric Surgery, Tbilisi Pediatric Private Clinic, Tbilisi, GEO; 5 Pediatrics, Tbilisi Pediatric Private Clinic, Tbilisi, GEO

**Keywords:** enteric duplication cyst, gastrointestinal congenital anomaly, pediatric surgery, small intestinal obstruction, surgical acute abdomen

## Abstract

Enteric duplication cysts are rare congenital malformations of the gastrointestinal tract that may present with a broad spectrum of clinical manifestations, ranging from incidental findings to life-threatening complications such as intestinal obstruction, volvulus, perforation, and gastrointestinal bleeding. Due to their nonspecific clinical presentation, preoperative diagnosis remains challenging.

We report the case of a four-year-old boy who presented with a three-day history of fever, diarrhea, progressive lethargy, and colicky abdominal pain. Initial clinical assessment suggested acute infectious gastroenteritis with dehydration. Laboratory investigations demonstrated elevated inflammatory markers, while abdominal ultrasonography revealed bowel wall thickening, mesenteric lymphadenopathy, and a cystic lesion associated with dilated bowel loops. Despite conservative treatment with intravenous fluids and symptomatic management, the patient's condition progressively deteriorated. He developed worsening abdominal pain, increasing abdominal distension, impaired passage of stool and flatus, and persistent fever. Repeat imaging demonstrated persistent bowel dilatation and multiple air-fluid levels consistent with mechanical intestinal obstruction. Emergency exploratory laparotomy revealed a large enteric duplication cyst arising from the mesenteric border of the small intestine. Partial small bowel resection and drain placement were performed. This case illustrates the diagnostic challenges posed by enteric duplication cysts presenting with symptoms mimicking acute gastroenteritis. Progressive abdominal distension and failure to improve with conservative treatment should prompt repeated surgical assessment and further imaging to exclude underlying mechanical obstruction.

## Introduction

Enteric duplication cysts are rare congenital anomalies of the gastrointestinal tract characterized by a smooth muscle wall and an epithelial lining resembling a segment of the alimentary tract [1,2]. Their estimated incidence ranges from approximately one in 4,500 to one in 10,000 live births [3,4]. Although duplication cysts may occur throughout the gastrointestinal tract, the ileum is the most common site of involvement [1,3,4].

Clinical presentation varies considerably according to lesion size, location, and associated complications [4,5]. Patients may present with abdominal pain, vomiting, gastrointestinal bleeding, intussusception, volvulus, bowel obstruction, perforation, or an abdominal mass [1,4-8]. Because symptoms are frequently nonspecific, preoperative diagnosis remains challenging [4,6,7].

We report a case of a large enteric duplication cyst presenting as progressive mechanical small bowel obstruction in a four-year-old child whose initial presentation closely mimicked acute infectious gastroenteritis.

## Case presentation

A four-year-old boy was brought to our Emergency Department with a three-day history of fever, diarrhea, and worsening abdominal pain. He was born at term following an uncomplicated pregnancy and delivery, weighing 3500 g. His medical history was otherwise unremarkable; he had reached all developmental milestones appropriately, had no chronic conditions or prior surgeries, and was fully immunized. No prior travel history was mentioned.

At the time of admission, the child weighed 13 kg. His illness had begun with a fever of 38.5°C and watery diarrhea occurring four to five times a day. However, his parents became concerned when he began showing signs of progressive lethargy, somnolence, and intense colicky abdominal pain, which prompted them to seek emergency care. Upon examination, he appeared weak, pale, and dehydrated. His temperature was 38.0°C, respiratory rate was 27/min, oxygen saturation was 98%, and blood pressure was 108/60 mmHg. Abdominal examination revealed diffuse tenderness, though no focal guarding or rebound was noted at that time.

Given the initial absence of classic signs indicating an acute surgical abdomen, the patient was evaluated by the pediatric surgery team. The working diagnosis remained consistent with acute infectious gastroenteritis. Conservative management was initiated, consisting of intravenous crystalloids, probiotics, and symptomatic support. However, the patient’s clinical course was complicated by a poor tolerance for oral rehydration.

Laboratory investigations performed upon admission are summarized below (Tables 1-3).

**Table 1:** Complete blood count, venous blood gases Hb: Hemoglobin; RBC: Red Blood Cell Count; WBC: White Blood Cell Count; ESR: Erythrocyte Sedimentation Rate; CRP: C-Reactive Protein; pH: Potential of Hydrogen, BE: Base Excess.

Parameter	Result	Reference Range
Hemoglobin	126 g/L	120-160 g/L
RBC	5.0 ×10¹²/L	4.0-5.2 ×10¹²/L
WBC	4.4 ×10⁹/L	4.5-13.5 ×10⁹/L
Platelets	358 ×10⁹/L	150-450 ×10⁹/L
ESR	38 mm/h	<20 mm/h
Neutrophils	77.6%	40-70%
Lymphocytes	16.1%	20-45%
Monocytes	6.3%	2-10%
CRP	51.4 mg/L	<5 mg/L
pH	7.33	7.35-7.45
Base Excess (BE)	-5 to -7 mmol/L	-2 to +2 mmol/L
Glucose	7.7 mmol/L	3.9-5.6 mmol/L (fasting)

**Table 2:** Stool test

Parameter	Result	Reference Range
Consistency	Semi-formed	Formed to semi-formed
Color	Brown	Brown
Mucus	Moderate amount	Absent
Leukocytes	8-10 / HPF	0-5 / HPF
Erythrocytes	2-4 / HPF	0-2 / HPF
Helminth eggs	Not detected	Not detected
Protozoa	Not detected	Not detected

**Table 3:** Initial ultrasound findings RLQ: right lower quadrant.

Parameter	Result	Reference Range / Normal Finding
Bowel wall	Thickened	Normal thickness (<3 mm when distended)
Mesenteric lymph nodes	Enlarged	Not enlarged (usually <5-8 mm short axis)
Free fluid	Small amount	None or trace physiologic fluid
RLQ cystic lesion	32 × 12 mm	No cystic lesion present
Dilated bowel loop	Adjacent to urinary bladder	No bowel dilatation
Appendix	Not clearly visualized	Normal appendix visualized or no secondary signs of appendicitis

Sixteen hours after admission, the patient demonstrated no significant clinical improvement. Fever persisted, and bowel movements remained frequent, although only small amounts of stool were passed. The patient also developed increasing difficulty passing flatus. The surgical team initially remained cautious because the clinical presentation and laboratory findings were suggestive of an acute inflammatory or infectious process rather than an acute surgical abdomen.

Abdominal pain progressively intensified and became the predominant clinical complaint. Persistent somnolence was noted; however, the child frequently awakened due to severe pain and irritability. Pain severity was assessed as 7/10 using an age-appropriate pain scale.

On repeat physical examination, the abdomen was markedly distended and diffusely tender. The patient exhibited significant discomfort and refused abdominal palpation because of the severity of the pain. Overall, the clinical picture suggested progression of the intra-abdominal process despite ongoing treatment.

Given the patient's persistent fever, worsening abdominal pain, and increasing abdominal distension, repeat laboratory testing was performed (Tables 4, 5).

**Table 4:** Repeat imaging findings, abdominal ultrasound

Parameter	Result	Reference Range / Normal Finding
Cystic lesion	32 × 15 mm	No cystic lesion present
Wall thickness	1.5 mm	Normal bowel wall thickness ≤ 3 mm
Bowel loops	Multiple dilated loops	No bowel dilatation
Air-fluid levels	Multiple Kloiber cups	Absent
Impression	Mechanical intestinal obstruction	No evidence of obstruction

**Table 5:** Repeat complete blood count, venous blood gases RBC: red blood cell; WBC: white blood cell; pH: potential of hydrogen; Na: sodium; K: potassium; Cl: chloride.

Parameter	Result	Reference Range
Hemoglobin	113 g/L	120-160 g/L
RBC	4.4 ×10¹²/L	4.0-5.2 ×10¹²/L
WBC	6.2 ×10⁹/L	4.5-13.5 ×10⁹/L
Platelets	270 ×10⁹/L	150-450 ×10⁹/L
Neutrophils	86.4%	40-70%
Lymphocytes	12.0%	20-45%
pH	7.35	7.35-7.45
Sodium (Na)	135 mmol/L	135-145 mmol/L
Potassium (K)	3.4 mmol/L	3.5-5.0 mmol/L
Chloride (Cl)	102 mmol/L	98-107 mmol/L

**Figure 1:**
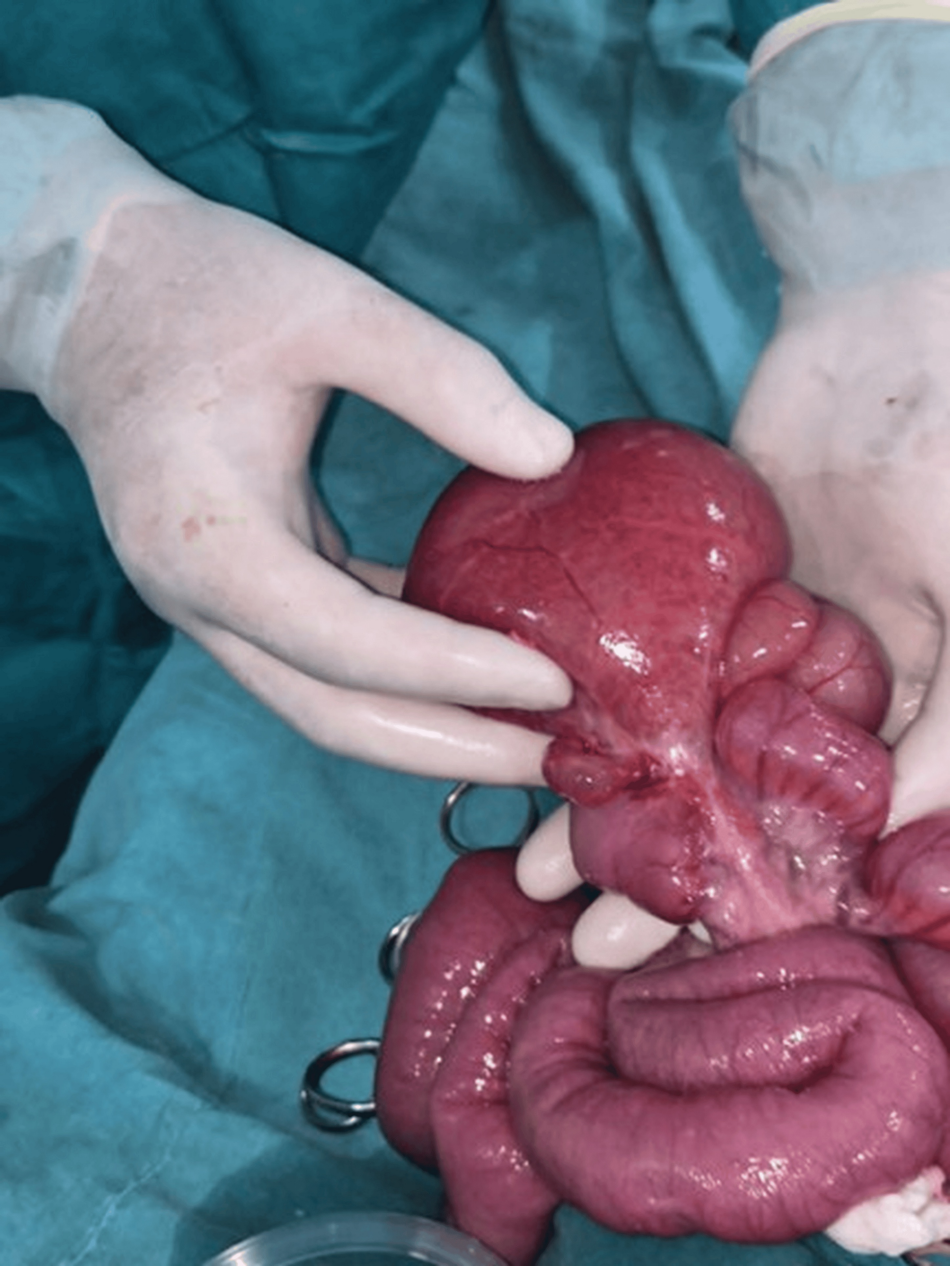
Intraoperative large cystic lesion arising from the mesenteric aspect of the small intestine with associated bowel dilatation

**Figure 2:**
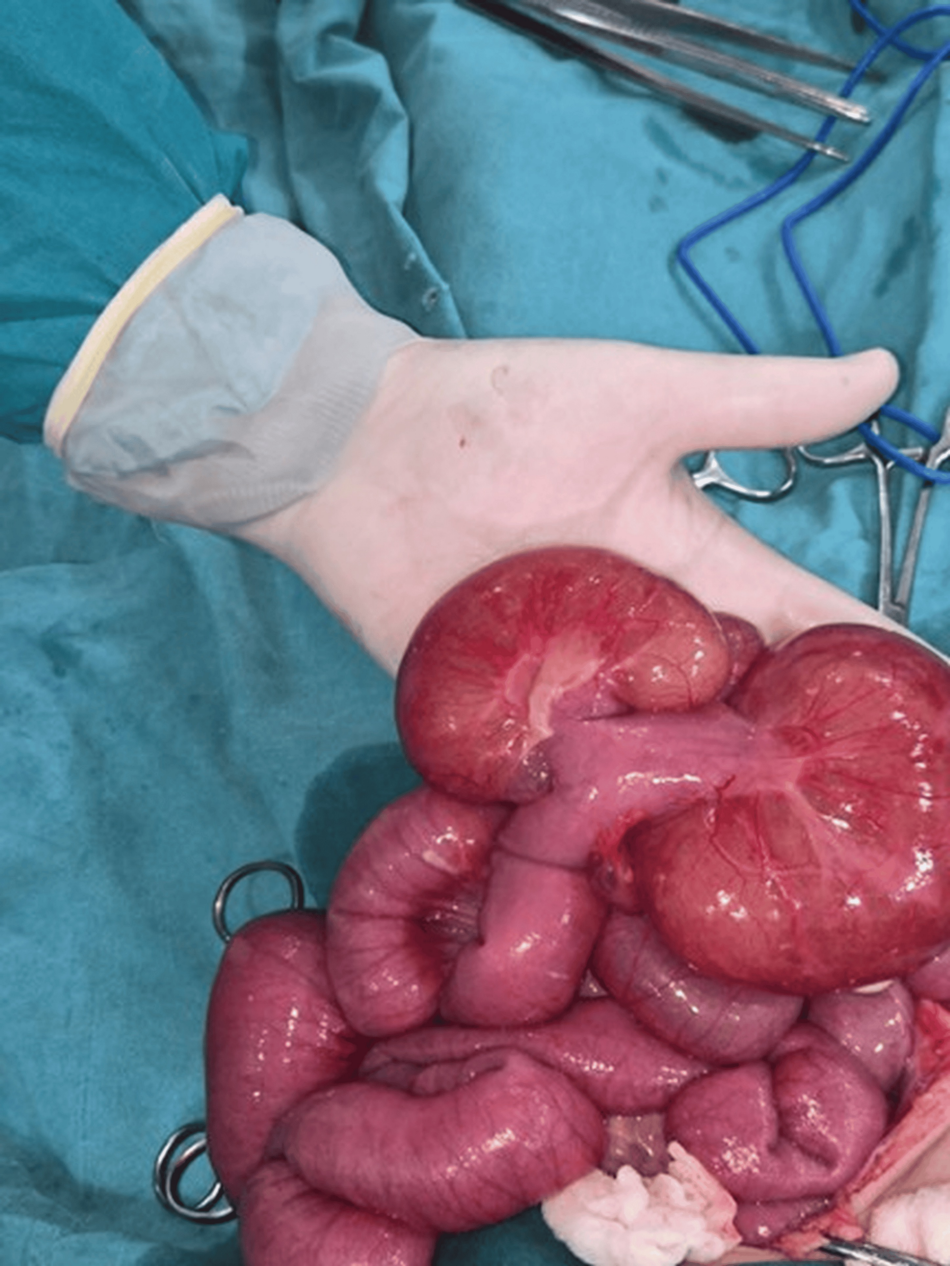
Closer operative view demonstrating the relationship between the duplication cyst and adjacent small-bowel loops.

Due to the progressive abdominal distension, worsening abdominal pain, persistent fever, and imaging evidence of mechanical intestinal obstruction, the patient underwent emergency exploratory laparotomy. A large enteric duplication cyst originating from the mesenteric border of the small intestine was identified intraoperatively. The affected bowel segment was resected, and an intra-abdominal drain was placed. Intraoperative findings are shown in Figures 1, 2. 

Histopathological examination confirmed an enteric duplication cyst of the small intestine measuring 20 × 25 mm. The cyst wall contained intestinal-type mucosa and a well-developed smooth muscle layer with chronic inflammatory changes. No malignant features were identified. Following an uneventful postoperative course, the patient was discharged 14 days after surgery. Following discharge, he was regularly followed in the outpatient setting. At follow-up visits, the child remained asymptomatic, demonstrated adequate oral intake, and did not report any episodes of abdominal pain. Bowel habits returned to normal, with a regular stooling pattern, and the patient exhibited a marked improvement in activity level compared with the preoperative period. No postoperative complications or recurrence of symptoms were noted. At the six-month follow-up assessment, the patient's mother reported that he had resumed age-appropriate physical activities, including playing football, and continued to feel well without any gastrointestinal complaints, indicating an excellent recovery and surgical outcome.

## Discussion

Enteric duplication cysts are rare congenital malformations of the gastrointestinal tract characterized by the presence of a well-developed smooth muscle wall and a mucosal lining resembling some portion of the alimentary tract [1,2]. They may occur anywhere along the gastrointestinal tract, although the ileum is the most frequently affected site [1,3,8].

The clinical presentation of enteric duplication cysts is highly variable and depends on the size, location, and associated complications of the lesion [4,7]. Common manifestations include abdominal pain, vomiting, gastrointestinal bleeding, a palpable abdominal mass, intussusception, and bowel obstruction [1,4,7,8]. However, diagnosis remains challenging because symptoms are often nonspecific and may mimic more common pediatric conditions [4,7].

In our patient, there was no history of recurrent abdominal pain or other symptoms suggestive of an underlying gastrointestinal anomaly prior to admission. The initial presentation, characterized by fever, watery diarrhea, and abdominal pain, was highly suggestive of acute gastroenteritis, a common condition in childhood. This clinical impression was further supported by stool microscopy demonstrating inflammatory changes and by elevated inflammatory markers. Notably, classical signs of an acute surgical abdomen were absent during the initial surgical evaluation; therefore, conservative management was considered appropriate.

Similar diagnostic challenges have been reported in the literature, where enteric duplication cysts initially presented as infectious or inflammatory conditions before the underlying surgical pathology became evident [4,7].

Despite adequate hydration and supportive treatment, the patient subsequently developed progressive abdominal distension, worsening abdominal pain, impaired passage of stool and flatus, and persistent fever. Repeat imaging demonstrated persistent bowel dilatation and multiple air-fluid levels (Kloiber cups), findings strongly suggestive of mechanical intestinal obstruction [8]. These changes prompted surgical exploration. Intestinal obstruction is a well-recognized complication of enteric duplication cysts and has been described as the presenting feature in several pediatric case series [4,7,8]. Although our patient's presentation initially mimicked acute gastroenteritis, the subsequent clinical course was more consistent with evolving intestinal obstruction secondary to the duplication cyst.

Ultrasonography remains the preferred initial imaging modality in pediatric patients with suspected enteric duplication cysts [1,4]. Typical findings include a cystic lesion demonstrating the characteristic double-wall or double-layer sign [1,7]. In our case, this specific sonographic feature was not reported; instead, only a cystic lesion and dilated bowel loops were identified. Establishing the preoperative diagnosis proved particularly challenging, as the initial clinical and radiological findings favored an infectious process rather than an acute surgical abdomen. The presence of diarrhea, inflammatory changes, and bowel dilatation further complicated the diagnostic assessment [4,7]. Although additional imaging studies such as computed tomography or magnetic resonance imaging were considered, the patient's rapidly deteriorating clinical condition ultimately led to the decision to proceed with exploratory laparotomy, through which the diagnosis was established [4,6,7]. In this case, exploratory laparotomy served as both the definitive diagnostic and therapeutic intervention.

Surgical excision remains the treatment of choice for symptomatic enteric duplication cysts [1,2,4-6,8]. Complete resection is generally recommended because of the risk of recurrent symptoms, bowel obstruction, bleeding, perforation, and, rarely, malignant transformation [4-6]. In the present case, partial small bowel resection was required because of the close anatomical relationship between the lesion and the adjacent bowel wall, a finding that has been described in previous reports [4,8].

This case highlights the importance of maintaining a high index of suspicion for underlying surgical pathology in children presenting with apparent gastroenteritis who fail to improve with appropriate conservative management [4,7,8]. Progressive abdominal distension, worsening pain, persistent fever, and impaired passage of stool and flatus should prompt repeat clinical evaluation and reconsideration of the differential diagnosis. Although enteric duplication cysts are uncommon, they should be considered among the differential diagnoses of children with suspected acute surgical abdomen, particularly when the clinical course is atypical or symptoms progressively worsen despite supportive treatment.

## Conclusions

Enteric duplication cysts can convincingly mimic common pediatric conditions, leading to significant diagnostic delays. When a child presents with apparent gastroenteritis but fails to improve with conservative management, surgical pathology must remain on the differential. Persistent fever, progressive abdominal distension, and cessation of bowel movements are warning signs that demand prompt reassessment and early surgical consultation. Early surgical consultation and repeat imaging in non-improving patients remains the cornerstone of successful management and prevents complications including obstruction, perforation, and the need for more extensive resection.
